# Characteristics of external loads of Hockey5s associated with the new version of U16 youth field hockey competition

**DOI:** 10.1038/s41598-023-32281-5

**Published:** 2023-03-28

**Authors:** Jan M. Konarski, Marcin Andrzejewski, Marek Konefał, Paweł Chmura, Mateusz Skrzypczak, Frantisek Zahalka, Tomas Maly, Robert M. Malina

**Affiliations:** 1 Theory of Sports Department, Poznań University of Physical Education, Krolowej Jadwigi 27/39, Poznan, Poland; 2 Faculty of Methodology and Recreation, Poznań University of Physical Education, Poznan, Poland; 3 Department of Biological and Motor Sport Bases, Wroclaw University of Health and Sport Sciences, Wrocław, Poland; 4 Department of Team Games, Wroclaw University of Health and Sport Sciences, Wrocław, Poland; 5 Faculty of Physical Education and Sport, Charles University, Prague, Czech Republic; 6 Professor Emeritus, Department of Kinesiology and Health Education, University of Texas at Austin, Austin, USA; 7 School of Public Health and Information Sciences and Department of Anthropology, University of Louisville, Louisville, KY USA

**Keywords:** Developmental biology, Health care

## Abstract

External workloads associated Hockey 5 s, the new version of youth field hockey, were evaluated in 31 elite U16 male field players (15.4 ± 0.7 years) from three national teams. Mixed-longitudinal observations for the 31 players provided complete data for 33 forwards and 43 defenders. Activities of the players during games were monitored with the GPSports SPI Elite System with a sampling frequency of 10 Hz and were analysed with GPSports Team AMS (version R1 2015.14, Australia). Observed variables did not differ between forwards and defenders, and the three periods of play were differentiated only by maximal speed in the second and third periods. The greatest distances covered were in speed zone 3 (10.0–15.9 km h^−1^; 35.5–38.2%) and the smallest in speed zones 4 (16.0–22.9 km h^−1^; 14.8–15.6%) and 5 (> 23 km h^−1^; 0.4–1.4%). The trends indicated high intensity levels for the entire match and by position and periods. Active time of forwards and defenders accounted for about one-half of a game’s duration (~ 15.7 of 30 min). Overall, the Hockey 5s format was highly demanding of players and included relatively short intervals for recovery. The results emphasize the need for preparation that includes specific mixed anaerobic and aerobic training and also the importance of recovery during breaks.

## Introduction

In an effort to attract, recruit and retain participants, many sports have modified traditional playing formats and in so doing introduced several new disciplines, e.g., beach volleyball and handball, or a smaller playing area with a reduced number of players, e.g. small-sided soccer. Such modifications and associated changes in official rules likely influenced the motor, physiological and psychological demands on players, e.g., variation in sport-specific skills, demands of training and competitions, and playing strategies. In addition, external loads vary among sports and sport formats^1–4^ and with player positions^5^.

A small-sided format of field hockey for U16 players, labeled “Hockey 5’s”, was introduced at the 2014 Youth Olympic Games^6^. The modified format reduced the number of players from 11 to 5 (including the goalkeeper, who could be substituted by a field player), decreased pitch size (from 91.40 × 55 m [5027 m^2^] to 48 × 31.76 m [1524.5 m^2^]), added boards (25 cm high) around the pitch, and reduced playing time from 2 periods of 35 min to 3 periods of 10 min with breaks between periods. A questionnaire survey of 170 Japanese youth field hockey players noted that awareness of the modified format was quite low, but two features of Hockey 5’s were favourably noted—the possibility of shooting from anywhere on the pitch and of using the wall for passing or shooting^7^.

Data addressing loads associated with the new Hockey5s format among youth players are limited. A preliminary study of 10 players 15–16 years noted that the internal and external loads associated with the Hockey5s format could be characterized as heavy exercise with an emphasis on speed and a need for quick recovery between spurts of intensive activity; the loads also differed between defenders and forwards^16^. Although limited to 10 players, the results implied a potential need to modify traditional training methods to better prepare youth to accommodate the aerobic and anaerobic demands of the new format. The preliminary observations also implied a need for more detailed assessment of external loads associated with the modified format among youth players and by inference a need to assist coaches in the development of appropriate training protocols. In this context, the purpose of this study was to evaluate the external loads associated with Hockey5s competitions among U16 players by field position and period of play, and relative to active playing time during a game.

## Methods

### Ethics

The study was in accord with the Declaration of Helsinki and was approved by the Human Ethics Research Committee of Karol Marcinkowski Medical University in Poznań (Poland) (No. 701/17). The study was also approved by the Polish Field Hockey Association and coaches of teams who represented other national associations. The research was conducted at the Poznań University of Physical Education, Theory of Sport Department, in cooperation with Charles University (Prague, Czech Republic).

### Subjects

The sample included 31 male field players (forwards, defenders) who participated in several competitive Hockey5s games over a two day tournament. All players and their parents/legal guardians, and coaches of their respective teams were informed of the procedures, benefits and potential risks of the tournament and assessment protocols, and provided signed informed consent on an institutionally approved form prior to the tournament. The forms were collected by the coaches of the respective teams.

The study was conducted during the Cup of 3 Nations International Hockey5s Tournament at the Olympic Center COS-OPO in Wałcz (Poland), which included teams from Poland (organizers), Switzerland and Austria. The weather during the two-day tournament was sunny with some clouds and a temperature of about 20 °C.

The playing surface was an artificial grass court 48 m × 31.76 m (total area 1524.5 m^2^) with a 25 cm high board built into the perimeter. Following official specifications, the court surface was watered prior to each game. Each team had four field players and a goalkeeper on the court; unlimited substitution was permitted within a specified area of the court in front of each team’s bench. Matches included three 10 min periods with an interval of 3 min between periods, consistent with rules specified for the Youth Olympic Games and the qualifying tournament. The organization of the tournament and the playing court were officially accepted by the FIH and were consistent with the relevant rules^8^. Coaches generally retained field players on the court during games for intervals of approximately 3–4 min and then changed the entire group. The pattern of substitution, however, varied with game situation and individual needs. The present analysis was limited to the period of active play.

The 31 players comprising the sample were members of the three U16 national teams which were actively training for the 2017 Euro Hockey5’s Championship, and included only players for whom complete data were available: Austria (n = 10), Poland (n = 12) and Switzerland (n = 9). Data were incomplete for one player, and seven goal keepers were excluded. Over the course of the two days of competitions, the three coaches selected the players for each game. As such, each of the 31 players comprising the sample participated in a variable number of games: one participated in 4 matches, 17 in 3 matches, 8 in 2 matches, and 5 in 1 match). Complete observations were thus available for 76 observations, including 33 forwards and 43 defenders. The present analysis is based on the mixed-longitudinal sample of 76 observations of the 31 players.

Based on interviews with the coaches, the typical weekly training load prior to the tournament included three field-based training sessions of 60–80 min, one or two resistance training sessions that included upper body, lower body and total body protocols, and one or two matches. The players were students and as such participated in other activities in addition to training, including school physical education (45 min sessions, 2 or 3 times per week).

### Procedures

Three days before the competition, standing height was measured using a portable stadiometer to the nearest 0.1 cm (Holtain, Crosswell, Crymych, Pembs, UK), and body mass (barefoot, light clothing) was measured on a TANITA MC-780 scale (Tokyo, Japan). The TANITA scale included a bio-impedance analyser (BIA) with GMON software (version 3.2.8) which provided an estimate of muscle mass and fat mass for each player.

External loads of players were monitored during the competitions using GPS technology with a sampling frequency of 10 Hz (GPSports SPI Elite System, Canberra, Australia). The technology is valid and reliable, and increasingly used to monitor external workloads during competitions^9^. After each match, the GPS data were downloaded to the docking station and subsequently analysed using GPSports Team AMS (GPSports, version R1 2015.14, Canberra, Australia). The variables were extracted included total distance covered (TDC), high metabolic load distance (HMLD), maximal running speed (Speed_max_) and average speed (Speed_ave_). Based on the original software, five speed Zones were indicated: Zone 1: 0–5.9 km h^−1^; Zone 2: 6.0–9.9 km h^−1^; Zone 3: 10.0–15.9 km h^−1^; Zone 4: 16.0–22.9 km h^−1^; and Zone 5: > 23 km h^−1^. Sprint distance was calculated when speed exceeded 23.0 km h^−1^ (> 6.39 m s^−1^); this threshold was used in previous studies of elite field hockey^10^ and soccer^11,12^ players.

### Analysis

The data are presented as measures of volume in active time and intensity; the latter included data for the active time of each player (m min^−1^) and/or the number of sprints in active time (n min^−1^). All variables were checked for normal distribution (Shapiro–Wilk test) and homogeneity of variance (Levene’s test). Means, standard deviations and 95% confidence intervals (95% Cl) were calculated. A linear mixed model was used; the model identifies fixed effects (i.e., underlying trends of a particular component of the sample) and also the unexplained variance around the mean trend for the component due to random effects (i.e., the 76 observations for the 31 players). Player position and period of play were modelled as fixed effects, while individual players were modelled as random effects to account for the repeated measurements. When a significant effect was noted, it was followed by a post-hoc Bonferroni test. Statistical significance was set at p < 0.05. Partial eta squared (η_p_^2^) was also calculated and effect sizes were estimated as follows: ≥ 0.01 and < 0.06—small, ≥ 0.06 and < 0.14—medium, and ≥ 0.14—large. Statistical analyses were performed using STATISTICA ver. 13.1 software (StatSoft Inc., USA).

## Results

Descriptive statistics for chronological age, height, weight and estimated body composition of the 31 players are summarized in Table 1. The players had 7.2 ± 2.2 years of experience in the traditional format of field hockey and were on the respective national teams for 3.1 ± 1.3 years. Given the possibility of participating in Hockey 5’s, the players trained simultaneously in both the traditional and new formats.

**Table 1:** Characteristics of the 31 players: means (M), standard deviations (SD) and ranges.

	M	SD	Range
Age, years	15.4	0.7	13.7–16.3
Height, cm	174.2	8.2	154.7–190.0
Weight, kg	63.5	8.0	45.6–79.0
Muscle mass, kg	51.2	6.5	36.2–62.3
Fat mass, %	15.2	2.1	9.5–19.2

Descriptive statistics for the measures of volume (absolute) and intensity (per unit active playing time, i.e., external loads placed upon players per unit playing time during a match), are summarized in Table 2. Players were active for approximately one-half of the total duration of a match (15.7 ± 2.3 of 30 min over three 10 min periods).

**Table 2:** Means (M), standard deviations (SD) and 95% confidence intervals (95% CI) for indicators of volume and intensity (per unit playing time) of effort during U16 Hockey5s matches.

	M	SD	95%Cl	
Volume				
Total time, min	15.7	2.3	15.2	16.2
Distance total, m	2140	319.8	2067	2213
Sprint distance, m	21.7	16.2	18.0	25.4
HMLD, m	729	139.3	697	761
Intensity				
RTD, m min^−1^	138.3	15.9	134.7	141.9
RSD, m min^−1^	1.4	1.2	1.2	1.7
RHMLD, m min^−1^	47.4	8.8	45.4	49.4
Speed _max_, km h^−1^	25.3	17	24.9	25.7
Speed _ave_, km h^−1^	8.2	0.7	8.0	8.3

The absolute volume and relative intensity of physical effort among forwards and defenders are summarized in Table 3. None of the volume and intensity measures differed significantly by position.

**Table 3:** Means (M), standard deviations (SD) and 95% confidence intervals (95% CI) for indicators of volume and intensity (per unit playing time) of effort by player position (forwards, defenders) during U16 Hockey5s matches, and results of the linear mixed model analysis and estimated effect sizes (η_p_^2^).

	Forwards (n = 33)				Defenders (n = 43)				F	P	η_p_^2^
	M	SD	95%CI		M	SD	95%CI				
Volume											
Total time, min	15.6	2.1	14.9	16.4	15.7	2.5	15.0	16.5	0.01	0.987	0.001
Distance total, m	2148	265.0	2054	2242	2134	359.2	2023	2244	0.01	0.993	0.001
Sprint distance, m	16.7	15.7	11.1	22.2	25.5	15.8	20.7	30.4	0.84	0.401	0.139
HMLD, m	750	104.2	713	787	713	160.5	664	763	1.36	0.292	0.199
Intensity											
RTD, m min^−1^	140.3	18.5	133.7	146.9	136.7	13.5	132.6	140.9	0.09	0.774	0.015
RSD, m min^−1^	1.1	1.0	0.7	1.4	1.7	1.2	1.3	2.1	0.72	0.435	0.123
RHMLD, m min^−1^	48.9	8.7	45.8	52.0	46.2	8.7	43.5	48.9	1.02	0.357	0.159
Speed_max_, km h^−1^	24.9	1.8	24.2	25.5	25.7	1.5	25.2	26.1	0.40	0.552	0.072
Speed_ave_, km h^−1^	8.2	0.7	8.0	8.5	8.1	0.6	8.0	8.3	0.03	0.881	0.005

HMLD, high metabolic load distance; RTD, relative total distance; RSD, relative sprint distance; RHMLD, high relative metabolic load distance.

Corresponding comparisons of absolute volume and relative intensity in the total sample of players by period of play are summarized in Table 4. Only maximal speed expressed per unit time (speed_max_. km h^−1^) differed significantly by period of play (p = 0.004, medium effect size). Maximal speed per unit time decreased, on average, between the second and third periods.

**Table 4:** Means (M), standard deviations (SD) and 95% confidence intervals (95% CI) for indicators of volume and intensity (per unit playing time) of effort by period of play during U16 Hockey5s matches, and results of the linear mixed model analysis, post-hoc comparisons and estimated effect sizes (η_p_^2^).

	Period of play												F (sig.)	Post hoc	η_p_^2^
	First				Second				Third						
	M	SD	95%CI		M	SD	95%CI		M	SD	95%CI				
Volume															
Total time, min	5.3	1.0	5.1	5.6	5.3	1.3	4.99	5.60	5.1	1.5	4.7	5.4	0.83 (0.44)	–	0.017
Distance total, m	736	137.4	704	767	724	184.8	682	766	680	192.0	636	724	1.60 (0.21)	–	0.034
Sprint distance, m	7.1	7.9	5.31	8.9	7.9	8.7	5.9	9.9	6.7	9.6	4.5	8.9	0.48 (0.62)	–	0.010
HMLD, m	253	58.5	240	267	246	66.9	231	262	230	76.9	212	247	2.40 (0.10)	–	0.052
Intensity															
RTS, m min^−1^	138.6	14.2	135.3	141.8	137.2	14.3	133.9	140.5	139.0	40.1	129.9	148.2	0.55 (0.58)	–	0.016
RSD, m min^−1^	1.6	1.5	1.0	1.7	1.5	1.7	1.1	1.9	1.5	2.4	0.9	2.0	0.09 (0.91)	–	0.001
RHMLD, m min^−1^	48.2	10.9	45.8	50.8	47.3	10.9	44.9	49.8	46.5	13.9	43.3	49.7	0.38 (0.69)	–	0.009
Speed_max_, km h^−1^	23.8	1.9	23.4	24.2	24.1	2.0	23.6	24.5	22.4	2.2	22.9	23.9	3.36 (0.04)	P2 > P3	0.067
Speed_ave_, km h^−1^	8.3	0.8	8.1	8.8	8.2	0.9	8.0	8.4	8.1	1.0	7.9	8.3	0.65 (0.52)	–	0.014

HMLD, high metabolic load distance; RTD, relative total distance; RSD, relative sprint distance; RHMLD, high relative metabolic load distance.

**Figure 1:**
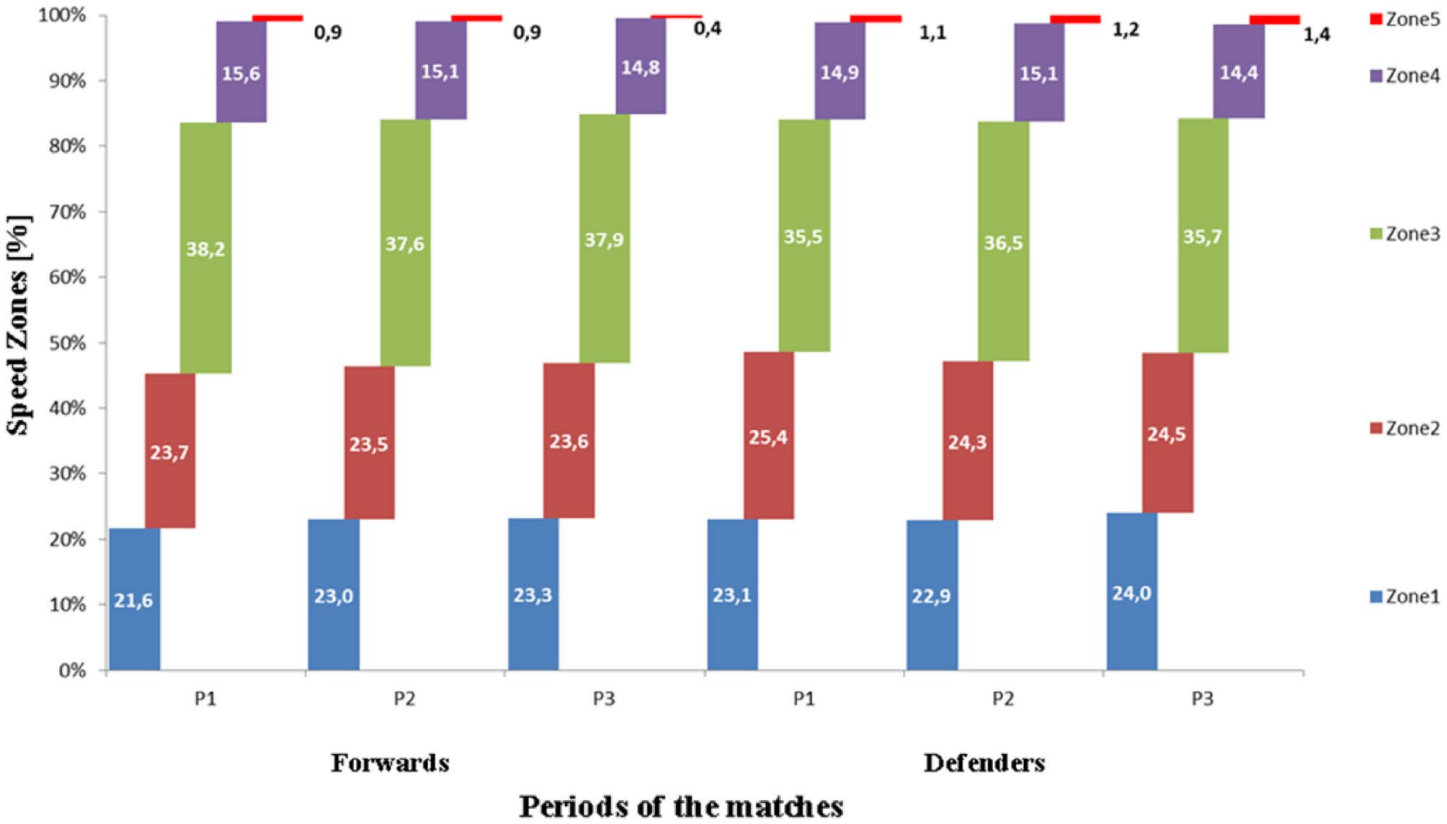
Relative distributions of ranges of running intensity (%) by player position and period of play [P1, P2, P3 = periods of the game; Zone 1: 0–5.9 km h^−1^; Zone 2: 6.0–9.9 km h^−1^; Zone 3: 10.0–15.9 km h^−1^; Zone 4: 16.0–22.9 km h^−1^; and Zone 5: > 23 km h^−1^].

Comparisons of indicators of volume and intensity among forwards and defenders within period of play are summarized in Table 5. None of the indicators of volume and intensity differed significantly by period of play among forwards and defenders. Nevertheless, the relative distributions of the five different running intensities (zones of speed) in matches by player position and period of play suggest several trends (Fig. 1). On average, the players covered the greatest distances in zone 3 (35.5–38.2%) and the smallest distances in, respectively, zones 4 (14.8–15.6%) and 5 (0.4–1.4%). Although differences by player position and among periods were not significant, the distributions highlighted the high intensity of a Hockey 5s format.

**Table 5:** Means (M) and standard deviations (SD) for indicators of volume and intensity (per unit playing time) of effort among forwards (n = 33) and defenders (n = 43) by period of play during U16 Hockey5s matches, and results of the linear mixed model and estimated effect sizes (η_p_^2^).

	Period of play						F (sig.)	Post hoc	η_p_^2^
	First		Second		Third				
	M	SD	M	SD	M	SD			
Forwards									
Volume									
Total time, min	5.3	0.9	5.3	1.1	5.0	1.5	0.26 (0.775)	–	0.011
Distance total, m	748	124.7	731	149	670	188.1	0.26 (0.776)	–	0.011
Sprint distance, m	6.4	7.1	6.8	8.3	3.5	6.3	0.18 (0.838)	–	0.006
HMLD, m	267	55.1	254	59.1	229	67.3	0.93 (0.402)	–	0.038
Intensity									
RTD, m min^−1^	141.3	13.5	137.4	14.8	142.3	51.1	0.59 (0.557)	–	0.029
RSD, m min^−1^	1.2	1.3	1.2	1.6	0.72	1.5	0.01 (0.995)	–	0.001
RHMLD, m min^−1^	50.9	10.5	48.0	9.8	47.7	14.0	0.75 (0.479)	–	0.033
Speed_max_, km h^−1^	23.7	2.0	24.0	2.1	22.4	2.1	2.10 (0.133)	–	0.075
Speed_ave_, km h^−1^	8.4	0.8	8.2	0.9	8.1	1.1	0.47 (0.631)	–	0.022
Defenders									
Volume									
Total time, min	5.4	1.1	5.3	1.5	5.1	1.5	0.96 (0.388)	–	0.033
Distance total, m	727	147.3	719	210	688	196.8	0.14 (0.328)	–	0.041
Sprint distance, m	7.6	8.6	8.7	9.0	9.1	11.0	0.02 (0.978)	–	0.001
HMLD, m	243	59.4	241	72.5	230	84.4	0.10 (0.342)	–	0.041
Intensity									
RTD, m min^−1^	136.5	14.6	137.1	14.0	136.6	29.5	0.12 (0.888)	–	0.004
RSD, m min^−1^	1.5	1.7	1.7	1.7	2.0	2.8	0.49 (0.618)	–	0.018
RHMLD, m min^−1^	46.2	10.9	46.8	11.7	45.6	14.0	0.73 (0.488)	–	0.029
Speed_max_, km h^−1^	23.9	1.9	24.1	2.0	24.1	2.0	0.03 (0.973)	–	0.001
Speed_ave_, km h^−1^	8.2	0.9	8.2	0.8	8.1	1.0	1.62 (0.206)	–	0.048

HMLD, high metabolic load distance; RTD, relative total distance; RSD, relative sprint distance; RHMLD, high relative metabolic load distance.

## Discussion

The characteristics of the external loads placed upon U16 players during active playing time in Hockey5s games by field position and period of play were considered. Two key observations were noted. First, playing time and external loads did not differ between forwards and defenders. Second, variation in external loads by period of play differed only for maximum speed of running in the total sample of players though not by position; the highest maximal speed occurred in the second period. The results likely reflect player preparation in the new format and implementation of the tactics designed by the coaches, including perhaps recognition of the strengths and weaknesses of the opponent during the first period, creating opportunities to gain an advantage combined with the high intensity during the second period, and maintaining the strategy but slowing the pace of the game due to increasing fatigue in the third period. The preceding, of course, reflects one possible sequence of events often used in team sport games, and requires confirmation with specific observations. Of note, the active time of forwards and defenders did not differ and approximated 50% of total match time (~ 15.7 min of 30 min), indicating the high intensity of effort required by the Hockey 5s format. These general observations merit further study as they have implications for player preparation, specifically the importance of match preparation and warm-up, the role of substitution during a match and the interval of waiting to the return to the field, and the importance of biological or psycho-physical recovery (and perhaps diet) after a match, and among other factors.

A study of running performance and game performance variables among professional Croatian national level soccer players (age: 23.9 ± 2.9 years) used a similar approach as the present study^5^. Although based on older players in a different sport, the authors emphasized the need to evaluate each position separately in the context of the specific demands or requirements associated with the respective positions. For example, the central midfielders in soccer covered the longest total distance and the longest distances jogging and running, whereas the lateral midfielders covered the longest distances in high-speed running and sprinting.

Results of the present study were generally consistent with preliminary observations of the Hockey 5s format in a sample of 10 Polish players who were, on average, older, taller and heavier than participants in the present study and played in only two games^13^. The present study sample was larger and represented three countries, and the majority of players participated in three games during the tournament. The present study also noted the high level of competitive effort required by the new format, which can be classified as heavy exercise relative to the assessment scale of Achten and Jeukendrup^14^.

Although studies considering external loads experienced by youth in the Hockey 5s format are not presently available, comparisons with other small-sided formats may provide insights. For example, a study of 16 soccer players (16.3 ± 0.6 years) participating in 4-on-4 soccer training matches noted continuous activity over 24 min of play on a small field (40 × 30 m, 1200 m^2^) with mini goals and no goalkeepers^15^. The players covered a distance of 2650 ± 18 m, while the distance covered in sprints was 65.4 m (2.5% of total distance). In contrast, the duration of a Hockey 5s game was longer (3 × 10 min), the total distance covered was less, 2140 ± 320 m (Table 2), but a considerably greater distance was covered in sprints, 21.7 ± 16.2 m (1% of total distance).

A study of adult players (27.4 ± 1.5 years) participating in a 4-on-4 free play soccer format on a smaller field (30 × 20 m; 600 m^2^) for four periods of 4 min duration noted a total distance covered of 2664 ± 237 m and 382 ± 56 m in sprints^16^; the distance covered in sprints accounted for about 14.5 ± 2.5% of the total distance. Although the observations noted a somewhat similar effort as noted in youth small-sided soccer and Hockey 5s matches, proportionally more sprints were observed in Hockey 5s games.

Studies of variation in workloads during small-sided competitions depend, of course, on the measurement systems and speed zone ranges used. The present study used the same protocol and speed zone ranges as used in previous studies of field hockey^10^ and soccer^11,12^.

In contrast to the preceding, results of the present study of Hockey 5s differed from observations of elite adult hockey players^17–20^. In theory, the activity profiles of youth and adult players and implicitly the physical demands placed on players should vary with level of skill and playing position at high levels of effort. This would also include the motor skills used in the macrocycles and tactical concepts essential for matches.^21,22^ Of interest, the observations of U16 players in the Hockey 5s format were not consistent with the preceding generalizations. Both forwards and defenders achieved high and similar intensity levels based on RTD, though other variables monitored during the matches differed. Although a systematic decrease in mean distance covered in minutes was noted across the three periods of play (Tables 4 and 5), the highest range of variability (based on standard deviations) was observed during the third period, which was the only period of play that showed below average distances covered for all players. Similar results were also observed in the 1st and 3rd periods for speed per minute, but the range of variation was greater in the 3rd period. Although differences were not significant, it is important to note that the HMLD was largely above average for all parameters and decreased systematically by period of play. Consistent with other variables, variation in HMLD was highest in the 3rd period. Forwards also achieved, on average, higher values than defenders for all variables considered (Tables 4 and 5), but the differences were not significant. Nevertheless, both forwards and defenders achieved high levels on the indicators of intensity of competitive effort. Trends noted in the present study thus suggest that the training of players for the Hockey 5s format should be more specific in order to facilitate better recovery during breaks within a period of play and between periods.

When comparing the results for RHMLD (m/min) with the most demanding interval of time during a soccer match (time duration 10 min) and Hockey 5s, U16 players in the latter format achieved higher values (forwards, 48.0 ± 8.7 m/min; defenders, 46.2 ± 8.7 m/min, Table 3) compared to field position soccer players (central defenders, 23.4 ± 3.6 m/min; full backs, 29.8 ± 4.5 m/min; midfielders, 29.2 ± 4.0 m/min; wide midfielders, 31.5 ± 6.6 m/min; and forwards, 26.6 ± 5.7 m/min)^23^. A subsequent report^24^ noted greater values of RHMLD with increasing surface dimension per player. In small-sided soccer games on different surface sizes (21.0 m^2^, 38.5 m^2^, 61.4 m^2^ and 73.7 m^2^), RHMLD values were lower among fullbacks for the four field sizes, respectively, 14.8 ± 6.8 m/min, 18.4 ± 6.5 m/min, 18.4 ± 7.6 m/min and 22.3 ± 6.3 m/min, compared to players in other positions. The surface dimension per player in Hockey 5s was larger (190.6 m^2^) compared with the small-sided soccer games, and may be a factor that contributed to the higher external loads for this modified format.

Of potential relevance, speed of sprint-skating decreased by 5–8% in the third compared with the first two periods of play among elite adult ice hockey players^25^, suggesting a potential role of fatigue. Neuromuscular fatigue also had a negative impact on specific skills^26^ and overall performances towards the end of a match^27^ among soccer players. It has also been suggested that neuromuscular fatigue may be a risk factor for muscle injury^28^.

The present study is not without limitations. The study considered the new, modified format of youth field hockey, Hockey 5s, and as such studies with which the present observations could be directly compared are limited. The study was also limited to external loads. Parameters indicative of internal loads, e.g., heart rate or lactate levels, in response to the demands of the Hockey 5s format need study.

The heights and weights of the sample of Hockey 5s players were, on average, within the range of other studies of field hockey players^29^, but information on their biological maturity status (skeletal age, pubertal status) was not available. Allowing for variation in methods of assessment, skeletal ages of soccer, ice hockey and roller hockey players indicate advanced maturity status among players 13–16 year of age and a significant number of players 15 and 16 years who are skeletally mature^29^.

As the players comprising the three teams represented national selections, it is likely that their physical and physiological fitness, general motor proficiency and specifically proficiency in field hockey skills were at a very high level. By inference, generalizing observations of the present study to recreational level field hockey and other formats should be done with care.

Allowing for the limitations and perhaps others, results of the present study are potentially important in the context of subsequent research addressing the U16 Hockey5s format. It must be initially recognized that the players are largely late adolescent youth, some of whom are still adjusting to the physical, physiological and behavioral changes and demands associated with the growth spurt and sexual maturation. Specific to the new format, the effort expended by youth players in the Hockey5s tournament games can be characterized as heavy exercise, which highlights the need for careful preparation of youth players to accommodate the aerobic and anaerobic demands of the new format, i.e., high levels of speed and speed-endurance. The new format also requires specific preparation for quick recovery over short intervals for breaks within a period and also between periods. Variation in responses of forwards and defenders to the intensity of new format also merit attention.

### Practical applications

The Hockey 5s format is still relatively new and data addressing the demands of the format are limited. Nevertheless, results of the present study suggest a need for modifications of training protocols to accommodate the demands of the new format per se and the specific demands associated with player position, i.e., forwards and defenders. The limited data suggest that training protocols and associated research need to address player recovery during rest intervals within periods of play, between periods and also post-match. The limited data indicate a need for adequate aerobic preparation that supports the body’s recovery systems and builds a more efficient metabolism. Such preparation should facilitate persistence of the adolescent athlete for a longer interval under oxygen debt conditions and contribute to more efficient participation during competitions.

The behavioral demands of tournaments in which youth participate in several games over relatively short intervals also merit attention, specifically in the context of overall player well-being. Careful observations of the reactions of youth players to competitions in the new format should aid trainers and coaches in adjusting or modifying training protocols with the goal of appropriate and efficient preparation for and adaptation to such competitions. Although beyond the scope of the present study, efforts are also needed to integrate tactical and match-specific preparations with the physiological and behavioral demands of the Hockey 5s format.

## Data Availability

Correspondence and requests for materials should be addressed to J. M. K.
